# Supplementary material for: Mental flexibility assessment: A research protocol for patients with Parkinson’s Disease and Anorexia Nervosa
Source: PLoS One. 2023 Dec 20;18(12):e0293921. doi: 10.1371/journal.pone.0293921 (PMC10732438; doi:10.1371/journal.pone.0293921)
Supplement: Attachment — Submitted filename: Response to the reviewers.docx [file pone.0293921.s001.docx]

Dear reviewer, we would like to assure you that we have diligently reviewed and revised our manuscript to ensure that it complies with all the specified style guidelines, including proper file naming conventions. We have made the necessary adjustments to the file names in accordance with PLOS ONE's requirements.

As requested, we added section in Competing Interests:

“The authors have declared that no competing interests exist”.

All track changes in the manuscript are in green.

**Reviewer#1:**

**Reviewer #1:** The authors aim to develop an assessment framework for patients with AN and PD. While the goal is of great interest and clinical importance, there are some issues with the manuscript that should be resolved prior to publication.

**Response:** We would like to extend our sincere gratitude to Reviewer 1 for their insightful comments and constructive feedback. Their thoughtful evaluation has significantly contributed to the enhancement of our manuscript, and we genuinely appreciate their time and expertise in reviewing our work. Their valuable suggestions have played a crucial role in shaping the clarity and robustness of our research. We look forward to incorporating their recommendations into the revised manuscript, thereby improving the overall quality of our study.

**Reviewer 1:** There is no description of how the data from the different measurements is planned to be aggregated in a flexibility score or multiple flexibility subscores.

**Response:** We appreciate Reviewer #1's observation regarding data aggregation. We intend to aggregate data from different measurements into flexibility scores as follows:

Cognitive Flexibility: We plan to aggregate cognitive flexibility data by creating a composite score based on self-report data. This composite score will be derived through an exploratory data analysis (EDA) approach using linear principal component analysis (PCA), which identifies common dimensions among various cognitive flexibility tests, allowing for the aggregation of similar items from different scales. The result will be a weighted average score. We describe in-depth into 502-510:

*“For assessing Cognitive Flexibility, which involves a diverse array of measurements, we plan to construct a composite score solely relying on self-report data. This composite score will be derived through an Exploratory Data Analysis (EDA) approach employing linear principal component analysis (PCA). By applying PCA, we will extract common underlying dimensions from the various cognitive flexibility self-reports (e.g CFI, CFS, CCFQ, FREE, Dflex) allowing us to aggregate similar items from distinct scales into a weighted average score (Fig. 4)”.*

**Reviewer 1:** There is no description of any statistical analysis planned. Thus, I cannot determine whether sample size calculation is valid.

**Response:** We apologize for the oversight in the initial manuscript. We plan to conduct a comprehensive statistical analysis, which will include a 2x2 univariate analysis of variance (ANOVA) model to assess the effects of group membership (patients vs. controls) and time (T0 vs. T1) on cognitive and affective flexibility measures. This model will allow us to examine both main effects and interaction effects.

We describe in-depth into 502-563, we also added images to describe the experimental design and the data analysis.

*The statistical methodology employed in this study centers around a 2x2 univariate analysis of variance (ANOVA) model. In this model, the between-subjects factor encompasses the distinction between patients and controls, while the within-subjects factor is delineated by the acute phase upon hospitalization (T0) and post-rehabilitation (T1). The primary objective of adopting a univariate approach is to systematically assess the effects of both group membership and time on measures of cognitive flexibility and affective flexibility. Furthermore, we seek to investigate potential interaction effects that may exist between these factors (Fig.4). In the context of affective flexibility, we will consider transition indicators derived from physiological measuring (ECG, f-EMG, GSR, RSP) and analyzed by the Markov chain models. The choice of Markov chains as our analytical framework stems from their inherent capacity to capture probabilistic transitions between discrete states, a quality that aligns seamlessly with the nuanced shifts and fluctuations observed in affective experiences. By harnessing the capabilities of Markov chains, we dissect the temporal evolution of emotional states and elucidate the underlying patterns that define affective transitions. Our experimental design affords us the opportunity to calculate and evaluate affect variability across different affective transitions, which can subsequently be normalized and integrated into the Markov chains. Initially, we will calculate the reciprocal ratio between the absolute mean and variability of the signal standardized variability measures based on the inverse of Noise to Signal. The standardized variability index is computed for each transition, considering the 30-second interval between blocks (120''+-15'') (Figure 5). However, it is also necessary to consider state indexes for each block in the Markov matrix, specifically the transition of state e.g. A to itself, called state trait transition. State trait transitions represent the probability of remaining in the same emotional state, and in the Markov Matrix, they are represented as the main diagonal. These state trait indexes are determined during the central 30 seconds of each affective block (from 45 to 75 seconds) Within the Markov matrixes, we will relativize the prior transition variability indexes, to experiment the probability of passing from one affective state to another. Normalization of transition indexes is required because the sum of transitions in Markov matrices is 1. By doing so, we effectively capture the probability of transitioning between and into blocks, empowering to uncover the intricacies of affect dynamics in both music's auditory realm and images' visual domain.*

*From each subject's data and for each physiological signal, we extracted and analyzed only the main-diagonal indexes, denoted as state trait indexes (AA-BB-CC-DD), which convey the subject's probability of remaining anchored in a specific affective state (stability).*

*This meticulous methodology allows us to rigorously assess and understand the dynamics of affective experiences through the lens of Markov chains, offering valuable insights into the temporal evolution of emotional states in our experimental context.*

*Our anticipated outcomes encompass the following hypotheses:*

*1.	For participants within the control group, we hypothesize that there will be no statistically significant differences between the scores obtained at T0 and T1, both for measures of cognitive flexibility and affective flexibility. This expectation suggests that individuals in the control group are likely to maintain stable levels of cognitive and affective flexibility over time.*

*2.	Conversely, we expect an interaction effect between group membership (patients) and time (T0 to T1). This interaction effect signifies our hypothesis that cognitive flexibility measures will exhibit improvement over time among patients, leading to concurrent enhancements in affective flexibility. In particular, motor rehabilitation could have a significant impact on executive function, related to mental flexibility, while cognitive-behavioral therapy of individuals with AN could have an impact on both executive function and emotional flexibility.*

*In essence, our statistical analysis anticipates that patients will demonstrate increased cognitive and affective flexibility throughout the study period.*

*These hypotheses provide a clear framework for our statistical analysis and offer valuable insights into the expected outcomes of this multifaceted assessment of flexibility.*

**Reviewer 1:** While the selection of psychological questionnaires and tests is well described and seems reasonable, there is a lack of justification for the biochemical markers or how these may relate to flexibility.

**Response:** We acknowledge the concern regarding the justification for including biochemical markers in our study. The selection of biochemical markers such as interleukin-6 (IL-6) and cortisol is motivated by the growing body of research suggesting their potential relevance to psychological and affective states. IL-6, for instance, has been associated with mood disorders and stress responses, and cortisol is a well-established stress hormone. By including these markers, we aim to explore potential connections between the biochemical and affective dimensions of flexibility, which could provide valuable insights into the underlying mechanisms of these disorders (Friederich & Herzog, 2011; Halls et al., 2023; Mora-Maltas et al., 2023; Sarrar et al., 2011; Trapero & Cauli, 2014).

We added this part into lines 218-238:

*Furthermore, this study aims to assess endocrinological parameters, including interleukin 6, cortisol, serotonin, catecholamines, and endorphins, in individuals diagnosed with eating disorders. These assessments will be conducted at three time points: the beginning of the rehabilitation program (T0), the midpoint of the rehabilitation program, and the conclusion of the rehabilitation program (T1). The selection of these parameters is based on their established correlations with cognitive and behavioral flexibility* (3,75)*. For example, previous studies have demonstrated that a temporary reduction in cerebral serotonin levels can impact the cognitive processing of negative feedback in the context of reversal learning* (Chui et al., 2008; Friederich & Herzog, 2011; Trapero & Cauli, 2014) *Moreover, the utilization of psychostimulants such as methylphenidate, which exert an influence on the activity of dopamine and noradrenaline, has been linked to enhanced attention but possible impediment to cognitive flexibility. In experiments conducted on rhesus monkeys administered therapeutic doses of methylphenidate, researchers have noted a potential negative impact on task-switching abilities* (Uddin, 2021)*. These findings suggest that increased attentional focus may be accompanied by a reduction in cognitive flexibility. Furthermore, it has been observed that the striatal cholinergic systems play a role in influencing behavioral flexibility. Studies conducted on humans using proton magnetic resonance spectroscopy have revealed that a decrease in choline levels within the dorsal striatum is linked to a reduced number of perseverative trials during reversal learning. Previous studies have demonstrated that an excessive expression of IL-6 has the potential to cause cognitive dysfunction by negatively affecting neurotransmission in brain regions that regulate cognitive processes, including the hippocampus and prefrontal cortex. The dysregulation of neurotransmission has the potential to impair cognitive flexibility by causing difficulties in set-shifting and attention* (Chui et al., 2008; Friederich & Herzog, 2011; Halls et al., 2023; Sarrar et al., 2011; Trapero & Cauli, 2014)*.*

**Reviewer 1:** It is not stated how IL-6 and cortisol are planned to be analyzed.

**Response:** Thanks for your valuable comment. We add this part in lines 485-490.

**“***All endocrinological measures are analyzed in IRCCS Auxologico Piancavallo. Patients will be fasted in the morning between 7.30 and 8.30 am with a sample of 2 serum tubes and 3 plasma tubes for the dosage of interleukin 6, cortisol, serotonin, catecholamines and endorphins. The samples will be centrifuged and aliquoted at -80 degrees Celsius in order to preserve the analytes that will be dosed. A kit from the company IBL-International called an ELISA will be used to measure the dosages of serotonin, catecholamines, and endorphins*”.

**Reviewer #2:**

The manuscript describes the planned "IntellEGO" study, funded by the Italian Ministry of Health.
The manuscript in its present form describes a very interesting and timely study and is suitable for PLOS ONE from my perspective. Additionally, it presents the rationale behind the planned research, so that besides the description of the planned project, the character of an "opinion" and "review" paper emerges.
The topic is cognitive flexibility in Parkinson's disease and anorexia nervosa, which are also the study populations of the studies planned. The manuscript, written by many very experienced authors, has an unusual, refreshing writing style (e.g. "there is a ton of evidence" (line 461), with several unexpected turns in the flow. While the topic is exciting and the study relevant, some small changes could make the manuscript significantly more successful:

**Response:**

Thank you for taking the time to thoroughly evaluate our manuscript outlining the "IntellEGO" study, which is supported by funding from the Italian Ministry of Health.

We appreciate your kind words regarding the relevance and timeliness of the study, and we are pleased to hear that you find it suitable for publication in PLOS ONE. We also acknowledge your observation that the manuscript takes on elements of an opinion and review paper due to its inclusion of the rationale behind the planned research.

The focus on cognitive flexibility in Parkinson's disease and anorexia nervosa is indeed central to our investigation, and we are grateful for your recognition of this. We revised the writing style to make the text more comprehensible.

**Reviewer 2:** I would suggest checking whether the following information can be added:
- The manuscript contains no information on the planned statistical analysis, which is unusual for a protocol paper.

**Response:** Thank you for your feedback. We acknowledge the omission of detailed information on the planned statistical analysis in the initial manuscript. In the revised version, we will provide a comprehensive section detailing the planned statistical analysis. This will include information on the statistical tests and methods that will be used to analyze the data, as well as how we will handle variables of interest and control for potential confounding factors. We appreciate your suggestion to enhance the manuscript's clarity in this regard.

We added this part into lines 502-563

*The statistical methodology employed in this study centers around a 2x2 univariate analysis of variance (ANOVA) model. In this model, the between-subjects factor encompasses the distinction between patients and controls, while the within-subjects factor is delineated by the acute phase upon hospitalization (T0) and post-rehabilitation (T1). The primary objective of adopting a univariate approach is to systematically assess the effects of both group membership and time on measures of cognitive flexibility and affective flexibility. Furthermore, we seek to investigate potential interaction effects that may exist between these factors (Fig.4). In the context of affective flexibility, we will consider transition indicators derived from physiological measuring (ECG, f-EMG, GSR, RSP) and analyzed by the Markov chain models. The choice of Markov chains as our analytical framework stems from their inherent capacity to capture probabilistic transitions between discrete states, a quality that aligns seamlessly with the nuanced shifts and fluctuations observed in affective experiences. By harnessing the capabilities of Markov chains, we dissect the temporal evolution of emotional states and elucidate the underlying patterns that define affective transitions. Our experimental design affords us the opportunity to calculate and evaluate affect variability across different affective transitions, which can subsequently be normalized and integrated into the Markov chains. Initially, we will calculate the reciprocal ratio between the absolute mean and variability of the signal standardized variability measures based on the inverse of Noise to Signal. The standardized variability index is computed for each transition, considering the 30-second interval between blocks (120''+-15'') (Figure 5). However, it is also necessary to consider state indexes for each block in the Markov matrix, specifically the transition of state e.g. A to itself, called state trait transition. State trait transitions represent the probability of remaining in the same emotional state, and in the Markov Matrix, they are represented as the main diagonal. These state trait indexes are determined during the central 30 seconds of each affective block (from 45 to 75 seconds) Within the Markov matrixes, we will relativize the prior transition variability indexes, to experiment the probability of passing from one affective state to another. Normalization of transition indexes is required because the sum of transitions in Markov matrices is 1. By doing so, we effectively capture the probability of transitioning between and into blocks, empowering to uncover the intricacies of affect dynamics in both music's auditory realm and images' visual domain.*

*From each subject's data and for each physiological signal, we extracted and analyzed only the main-diagonal indexes, denoted as state trait indexes (AA-BB-CC-DD), which convey the subject's probability of remaining anchored in a specific affective state (stability).*

*This meticulous methodology allows us to rigorously assess and understand the dynamics of affective experiences through the lens of Markov chains, offering valuable insights into the temporal evolution of emotional states in our experimental context.*

*Our anticipated outcomes encompass the following hypotheses:*

*1.	For participants within the control group, we hypothesize that there will be no statistically significant differences between the scores obtained at T0 and T1, both for measures of cognitive flexibility and affective flexibility. This expectation suggests that individuals in the control group are likely to maintain stable levels of cognitive and affective flexibility over time.*

*2.	Conversely, we expect an interaction effect between group membership (patients) and time (T0 to T1). This interaction effect signifies our hypothesis that cognitive flexibility measures will exhibit improvement over time among patients, leading to concurrent enhancements in affective flexibility. In particular, motor rehabilitation could have a significant impact on executive function, related to mental flexibility, while cognitive-behavioral therapy of individuals with AN could have an impact on both executive function and emotional flexibility.*

*In essence, our statistical analysis anticipates that patients will demonstrate increased cognitive and affective flexibility throughout the study period.*

*These hypotheses provide a clear framework for our statistical analysis and offer valuable insights into the expected outcomes of this multifaceted assessment of flexibility.*

**Reviewer 2:** No information on the study site, is it a multicentre study?

**Response:** We appreciate this point. The “IntellEGO” study is conducted at a single study site, and we will explicitly mention this in the revised manuscript to clarify that it is not a multicenter study.

**Reviewer 2:** According to the ethics protocol, the project is designed as an “observational study”, but what effects do they expect from the therapy? Is the aim of the study to map therapy effects?

**Response:** Thank you for your important comment. We would like to clarify that our study is primarily designed as an observational study with the aim of assessing mental flexibility through a multifaceted assessment. This assessment considers the cognitive, affective, physiological, and endocrinological components of flexibility. The therapy mentioned in our study investigates whether mental flexibility is a trait (stable over time) or a state (modifiable through therapeutic intervention) variable.

Specifically, therapy is employed to assess whether mental flexibility, as measured by behavioral, physiological, and endocrine variables, can be influenced by therapeutic interventions, both of a cognitive-psychological and motor nature. The goal is to understand whether these therapies can modify mental flexibility in patients with Parkinson's disease and anorexia nervosa. This aspect is crucial in determining whether flexibility is amenable to changes through specific treatments.

In summary, our observational study aims to assess initial mental flexibility in patients and healthy controls and examine whether therapy can have a significant impact on this flexibility.

We deeply appreciate your observation and constructive feedback. In the revised version of our manuscript, we will make sure to provide even greater clarity on these research objectives to ensure a thorough understanding of our study's design and purpose.

Once again, we sincerely thank you for your valuable insights and commitment to improving the quality of our work.

We added this part into lines “473-476”:

*“The proposed rehabilitation is an element of clinical routine, and we need to understand whether and how mental flexibility, encoded in its cognitive-affective dimensions, is a trait or state dimension. Patients, after routine rehabilitation performed in the hospital, might have an improvement in the affective-cognitive components of MF* (Miles et al., 2020)*”.*

**Reviewer 2:** Clearer information on "about 40 subjects" with such a small number of cases (line 267) and "various population classes" in line 262.

**Response:** Thanks for the useful suggestion. We used “about” to consider eventually dropping out.

We added this line in 295-297.

*“In order to be more conservative and with an effect size of 0.34 and a statistical power of 0.95, we project about 40 subjects, considering eventually drop-out, for the patient and control groups, respectively”*

Lines 313-317

*“In order to be more conservative and with an effect size of 0.39 and a statistical power of 0.95, we project about 30 subjects, considering eventually drop-out, for the patient and control groups, respectively”*

**Reviewer 2:** A therapy program that is predominantly logopaedic and designed for flexibility of movement is described precisely. Please add whether in your opinion the psychological therapy contains elements of cognitive flexibility training or not. Do you expect effects of motor training on psychological flexibility? What statistical methods will be used to measure them?

**Response:** In our study, we believe that motor rehabilitation can directly impact the psychological flexibility of patients with Parkinson's disease and anorexia nervosa. This connection is based on the idea that the body and mind are inherently linked, and improving physical flexibility through motor training could have positive effects on mental flexibility.

Motor rehabilitation, focused on movement flexibility and coordination, may contribute to enhancing body awareness and the perception of one's physical capabilities. This, in turn, could positively influence the mental flexibility of patients, allowing them to better adapt to cognitive and behavioral challenges.

To measure this connection, we employ a statistical approach that examines correlations between improvements in motor flexibility and those in psychological flexibility. This will enable us to assess whether the effects of motor rehabilitation are reflected in improvements in mental flexibility.

Ultimately, our study aims to explore how motor rehabilitation may contribute to promoting better mental flexibility in patients with Parkinson's disease and anorexia nervosa, paving the way for new treatment modalities that take into account the interaction between body and mind.

We added this description into lines 468-477:

*“As a crucial step in assisting the refeeding process, the rehabilitation psychology program will be coordinated and supervised by the psychologist with the aid of the educator, with daily individual meetings.*

*Patients who can support physical activity will go through a standardized rehabilitation program: during these 4 weeks, they will be directed by a physiotherapist through exercises to improve their walking ability and endurance, and balance control an hour a day, from Monday through Saturday.* *The proposed rehabilitation is an element of clinical routine, and we need to understand whether and how mental flexibility, encoded in its cognitive-affective dimensions, is a trait or state dimension. Patients, after routine rehabilitation performed in the hospital, might have an improvement in the affective-cognitive components of MF (100)”*

And also into lines of Section Statistical Analysis (558-568)

*Conversely, we expect an interaction effect between group membership (patients) and time (T0 to T1). This interaction effect signifies our hypothesis that cognitive flexibility measures will exhibit improvement over time among patients, leading to concurrent enhancements in affective flexibility. In particular, motor rehabilitation could have a significant impact on executive function, related to mental flexibility, while cognitive-behavioral therapy of individuals with AN could have an impact on both executive function and emotional flexibility.*

*In essence, our statistical analysis anticipates that patients will demonstrate increased cognitive and affective flexibility throughout the study period.*

*These hypotheses provide a clear framework for our statistical analysis and offer valuable insights into the expected outcomes of this multifaceted assessment of flexibility.*

**Reviewer 2:** What is the role of the control group? In what way is it included in the statistical analysis? Is it really only matched by age as line 305 suggests? Are the study groups only analysed separately? Is a comparison of the disease groups planned?

**Response:** We appreciate your inquiry regarding the role of the control group and the statistical analysis plan. In the revised manuscript, we have provided a more comprehensive explanation of the control group's role, how it will be included in the statistical analysis, and any planned comparisons between the control group and the disease groups. We will clarify that age matching is just one aspect, and we will outline our approach to statistical comparisons and analyses involving all study groups.

We inserted those lines into Statistical analysis section 499-567

*“The control group plays a crucial role in determining whether mental flexibility is a trait or state characteristic among the subjects involved. It is included as a "between-subjects" measure in a 2x2 analysis of variance (ANOVA).*

*To ensure accurate comparability, subjects across different groups are matched for age and gender. Patient groups are analyzed separately due to significantly different levels of inflexibility.*

*In this initial stage of the study, our aim is exploratory. We are seeking to understand if there is potential to develop a multidimensional assessment of mental flexibility that can be applied across different pathologies.”*

**Reviewer 2:** Is medication recorded or controlled in the study? Is exercise controlled for before blood sampling?

**Response:** We appreciate your perceptive questions regarding the recording and monitoring of medication and physical activity in our study. Permit us to clarify our methodology with regard to these essential aspects of our research.

Patients diagnosed with Parkinson's disease and anorexia nervosa who are undergoing clinical supervision for the duration of their rehabilitation programs will be recruited for our study. This clinical supervision involves close monitoring of the patients' medical conditions, treatment plans, and overall progress. As a result, we will have access to comprehensive information regarding each patient's prescribed medications. Medication data, including dosage, frequency, and any adjustments made during the rehabilitation period, will be meticulously recorded in loco.

Regarding exercise control, we recognize its significance in ensuring the accuracy of our physiological and endocrine measurements. The rehabilitation exercise protocols will be carefully monitored and documented, on days other than the IAPS trial and the detection of endocrinological parameters. Blood sampling will be scheduled in a manner that controls for the effects of exercise, ensuring that samples are collected at optimal times to minimize potential interference from physical activity.

We added it in lines 493-494

*”Endocrinological detection will take place on days other than rehabilitation protocol.”*

**Reviewer 2:** The paper could benefit from more linguistic clarity.

**Response:** Dear reviewer, in response to your suggestion, we have conducted a thorough review and revision of the manuscript with a primary focus on improving linguistic clarity. We have clarified and refined the language throughout the document to ensure that the content is more accessible and comprehensible to our readers.

**Reviewer 2**. Especially the abstract could benefit from a restructuring, make it clearer that it is a protocol for a planned study, mention case numbers, title of the project and design already in the abstract.

**Response:** In light of the suggestions provided, we have undertaken a comprehensive restructuring of the abstract to enhance its clarity and explicitly convey its purpose as a delineation of a planned study protocol. We have incorporated pertinent information as per your recommendation.

The purpose of these revisions is to enhance the abstract's informational content and facilitate readers' comprehension of the study's nature and extent through this section.

We modify the abstract into lines 24-46

*Mental Flexibility oscillates between adaptive variability in behavior and the capacity to restore homeostasis, linked to mental health. It has recently been one of the most investigated abilities in mental and neurological diseases such as Anorexia nervosa and Parkinson's disease, studied for rigidity or cognitive inflexibility. Patients with anorexia nervosa have rigid cognitive processes about food and weight, which leads to restrictive eating and excessive exercise. People who struggle to adapt their cognitive processes and actions to change their diet and exercise habits may have a harder time recovering from the disorder. On the other hand, research suggests that Parkinson's disease patients may have cognitive flexibility impairments that impair their ability to perform daily tasks and adapt to new environments. Although of clinical interest, mental flexibility lacks theoretical liberalization and unified assessment.*

*This study introduces "IntellEGO" a protocol for a new, multidimensional psychometric assessment of flexibility. This assessment evaluates a person's authentic ability to handle daily challenges using cognitive, emotional, and behavioral factors. Since traditional assessments often focus on one domain, we aim to examine flexibility from multiple angles, acknowledging the importance of viewing people as whole beings with mental and physical aspects.*

*The study protocol includes two assessment phases separated by a rehabilitation period. T0, the acute phase upon admission, and T1, the post-rehabilitation phase lasting 15 days for Parkinson's patients and 4 weeks for eating disorder patients, will be assessed. Neuropsychological performance, self-report questionnaires, psychophysiological measures, and neuroendocrine measures will be collected from Anorexia Nervosa and Parkinson's Disease patients during each study phase. The objective of this procedure is to provide clinicians with a comprehensive framework for conducting meticulous assessments of mental flexibility. This framework considers emotional, cognitive, and behavioral factors, and is applicable to various patient populations.*

**-Reviewer 2:** "Rehabilitation" as a keyword?

**Response:** We appreciate the suggestion to include the keyword "rehabilitation" in our manuscript.

While it is true that patients undergo a rehabilitation process, our research focuses primarily on the assessment phase that patients undergo prior to and following the rehabilitation process. We believe that, given this specific focus, the chosen keywords would more accurately reflect the central themes and objectives of our research. These key terms highlight the phases of assessment that our study investigates, which ultimately contribute to a thorough comprehension of the rehabilitation process.

We hope this clarification clarifies our choice of keywords and aligns with the paper's primary focus. We greatly value your feedback and eagerly anticipate your continued guidance as we continue to revise our manuscript.

**Reviewer 2:** I don't understand figure 1 with this explanation, please add information what is meant by it.

We decided to combine Figure 1 and 2 to clarify the fact that cognitive flexibility, through self-reports and neuropsychological tests, and affective flexibility are two related components within the macro domain of mental flexibility. Using a comprehensive assessment of mental flexibility, allows us to fully investigate the two components, while also investigating their possible interactions.

**Reviewer 2:** Mention the title of the study ("IntellEGO") in the manuscript and in the abstract.
- Please introduce abbreviations beforehand (for example "AN" in line

**Response:** We have introduced the abbreviation “AN” and IntellEGO in the abstract.

**Reviewer 2:** The manuscript should be clear about the tense, avoid past tense like "collected" in line 305.

We replaced with the future tense.

**Reviewer 2:** Anorexia and anorexia nervosa should be separated in language and content (e.g. line 138), as they are separate concepts.

**Response:** Thank you very much for the valuable consideration, we only considered anorexia nervosa (AN).

**Reviewer 2:** Avoid "partially" in line 491. Funding of the project as part of the main manuscript?

**Response:** Thank you again for your careful review of our manuscript. We recognize that different institutions and journals may have varying conventions for the wording of funding acknowledgements, but the phrase "partially supported" is consistent with our institution's standard practice for acknowledging funding sources. It is intended to accurately convey the dual sources of financial support that contributed to the research presented in our paper.

Once again, we appreciate your thorough review and assistance in ensuring the accuracy and conformity of our manuscript with the journal's guidelines.

**Reviewer 2:** Little separation of content between introduction and discussion; if necessary, move the basic aspects from the discussion to the introduction.

**Response:** Thanks for your valuable comments. We revised introduction and discussion.

Introduction (lines 61-118)

*Mental Flexibility (MF) is a cross-cutting characteristic that is present in various cognitive-affective processes (1–3). This concept is commonly delineated in relation to cognitive, physiological, and emotional elements (3–10). Cognitive flexibility pertains to executive functions, specifically attention shifting and conflict monitoring (9,11–16). Psychological Flexibility (PF) or Emotional Flexibility (EF), instead, refers to the individual’s capability to change their behavioral patterns and their willingness to engage with emotions, thoughts, and sensations, including both desired and undesired private experiences (7,9,10). Psychological and emotional flexibility are often considered synonyms in scholarly literature, and for the purpose of this article, the term “emotional” will be employed (6). All components of cognitive and emotional flexibility exhibit a common characteristic, referred to as adaptive variability (3,17). Variability refers to the subject's ability to perceive, respond to, and comprehend events from multiple perspectives (8,10). Similarly, adaptability, which refers to the capacity to adapt to different contexts, situations, and challenges to accomplish a specific objective, is frequently connected with resilience and resourcefulness (18,19). Therefore, flexible behaviour entails the ability to adjust and modify one’s actions in response to changing situations and methodologies. In addition, previous studies have investigated the cognitive and emotional dimensions in conjunction with various constructs such as emotional intelligence, creativity, beliefs, and mentalizing capacities (11,12,14,20–23). In fact, the attribute of flexibility arises from the ability to adapt to social interactions, commonly referred to as emotional intelligence. Additionally, it stems from the ability to establish novel connections between ideas and beliefs, known as creativity. Furthermore, having a mindset oriented towards problem-solving, along with the capacity for beliefs and mentalizing, contributes to the development of flexibility. On the other hand, the unfavourable aspects of flexibility encompass rigidity and impulsiveness. Rigidity refers to the inclination to establish and maintain specific cognitive, emotional, or behavioral patterns, which are consistently utilized even in situations where these patterns are no longer advantageous. Rigidity is a psychological construct that has been characterized as the inclination to form and maintain consistent patterns of behavior (24,25). Impulsiveness is characterized by a proclivity to engage in rapid actions without due consideration of the potential outcomes, thereby constraining an individual's capacity to effectively adapt to novel circumstances or contemplate alternative strategies (26–28). Both problems can be indicative of specific mental health conditions as stated in the Diagnostic and Statistical Manual of Mental Disorders (DSM-5-TR) (24,25).*

*In this regard, both the DSM-5-TR and the U.S. National Institute of Mental Health have encouraged psychiatrists to evaluate flexibility in a dimensional manner rather than using categorical assessment. These research domain criteria (RDoC) approach recognizes that flexibility dimensions can transcend traditional diagnostic categories and encourages the integration of multiple levels of data, ranging from genomics to neural circuits to behavior and self-report (for example, using participant-filled questionnaires). This approach aims to understand the fundamental dimensions of functioning that encompasses the entire spectrum of human behavior. The "cognitive systems" domain of the RDoC matrix encompasses constructs such as "cognitive control," "emotion flexibility," and "working memory". These constructs consist of subconstructs, namely goal selection/performance monitoring and flexible updating, which exhibit a robust association with cognitive and emotional flexibility (29). Cognitive and emotional rigidity or inflexibility are commonly observed characteristics of many mental illnesses (3,16,30–35), specifically in clinical conditions that manifest in early life, such as Parkinson Disease (PD) and Anorexia Nervosa (AN). These traits have been extensively documented in the literature with numerous studies specifically examining their presence in PD (30,31,33,36,37) and AN (38–40).*

*On one side, recent research has established a correlation between motor stiffness in PD and executive processes (attention shifting and conflict monitoring), which are known to be affected by cognitive procedural rigidity. On the other side, AN is distinguished by its emotional rigidity and difficulties in transitioning between cognitive or affective states. Nevertheless, quantifying this emotional-cognitive rigidity remains unattainable at present. Occasionally, it is overlooked. The lack of evaluation in this context can be attributed to the inconsistent implementation of evaluation methodologies and the difficulties in precisely defining the concept of flexibility, which is currently in the process of being developed. To assess patients with PD and AN in terms of their emotional flexibility, our study describes a comprehensive assessment protocol. This method would enable a more precise restoration of people's behavioral adaptability and their ability to engage with the external environment (including people, emotions, and situations). The analysis of flexibility can be conducted across various dimensions in order to customize therapy according to the individual needs of the subject.*

**Reviewer 2:** Line 269ff. and 290ff. better as supplemental material.

**Response:** Thank you for this suggestion. We will move it to the supplementary section.
